# Sensory disturbances of buccal and lingual nerve by muscle compression: A case report and review of the literature

**DOI:** 10.4317/jced.52772

**Published:** 2016-02-01

**Authors:** Joaquín Alvira-González, Cosme Gay-Escoda

**Affiliations:** 1 DDS, MS. Master of Oral Surgery and Implantology. Faculty of Dentistry, University of Barcelona; 2 MD, DDS, PhD. Chairman of Oral and Maxillofacial Surgery. Director of the Master of Oral Surgery and Implantology. University of Barcelona Dental School. Coordinating reseacher of the IDIBELL Institute. Head of the Service of Maxillofacial Surgery, Teknon Medical Center. Barcelona, Spain

## Abstract

**Introduction:**

Several studies on cadavers dissection have shown that collateral branches of the trigeminal nerve cross muscle bundles on their way, being a possible etiological factor of some nerve disturbances.

**Case Report:**

A 45-year-old man attended to the Temporomandibular Joint and Orofacial Pain Unit of the Master of Oral Surgery and Implantology in Hospital Odontològic of Barcelona University, referring tingling in the left hemifacial región and ipsilateral lingual side for one year, with discomfort when shaving or skin compression.

**Discussion:**

Several branches of the trigeminal nerve follow a path through the masticatory muscles, being the lingual nerve and buccal nerve the most involved. The hyperactivity of the muscle bundles that are crossed by nerve structures generates a compression that could explain certain orofacial neuropathies (numbness and / or pain) in which a clear etiologic factor can not be identified.

** Key words:**Buccal nerve, paresthesia, idiopathic trigeminal sensory neuropathy.

## Introduction

There are several references in the literature regarding the neuropathy caused by compression. Among the best known are the sciatic syndrome (compression of nerve sciatica due to a bulging intervertebral disc or an occupying space tumor) or carpal tunnel syndrome (peripheral neuropathy caused when the median nerve is compressed within the carpal tunnel).

In the case of the trigeminal nerve, the mandibular branch can manifest these neuropathies, although it is difficult to relate the clinical findings with the anatomical disposition. Several cadaver dissection studies have shown the possibility of finding these anomalies in the anatomical path of collateral branches of the third branch of the trigeminal nerve, crossing the bundles of the masticatory muscles. The lingual and the buccal nerve are the most affected by this particularity, due to its proximity to the internal and external pterygoid muscles (1-5).

The aim of this paper is to present the case of a patient with sensory disturbances of the buccal and lingual nerve by muscle compression and a review of the literature on this type of neuropathy when is related to the orofacial region.

## Case Report

A 45-year-old man was seen in June of 2010 in the Temporomandibular Joint and Orofacial Pain Unit (Hospital Odontològic, Barcelona University, Spain) referring tingling in the left hemifacial region, including ipsilateral lingual side, for one year of evolution, accompanied by discomfort when shaving and skin compression. Once vascular disorders and central or peripheral tumors were excluded by several neurology services through the appropriate tests (computed tomography angiography, magnetic resonance imaging and laboratory blood test), the patient underwent thorough medical history and physical examination. The presence of local myalgia of the masticatory muscles (temporal and masseter) and pain in both temporomandibular joints (TMJ) in movement and at rest were observed.

**Figure 1:**
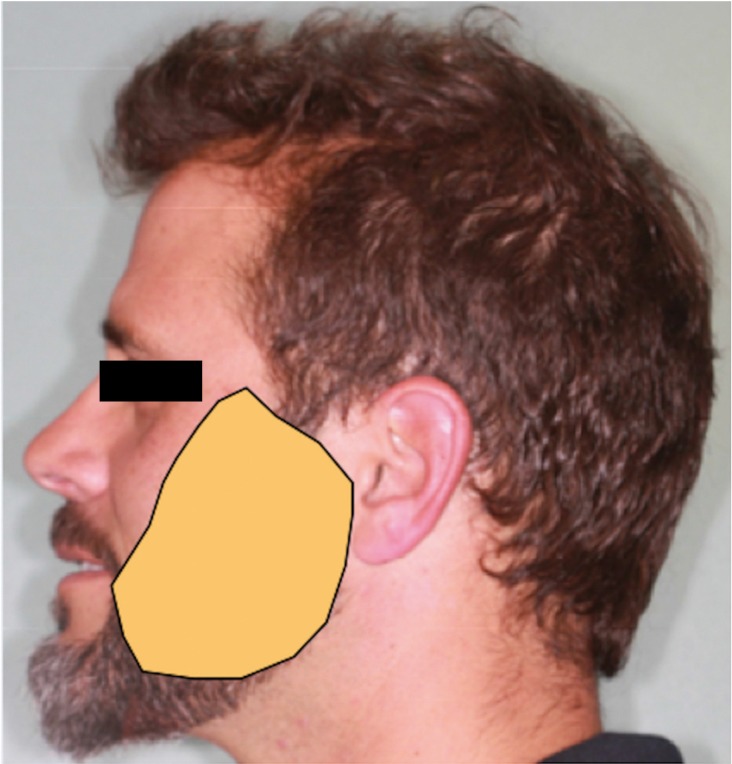
Clinical boundaries of the affected area.

The tingling sensation was located on the temporal zone at its upper limit, extending to the pinna on the posterior site and the corner of the mouth in its anterior localization, accompanied by a transient paresthesia of the left side of the tongue (Fig. 1). It was especially important when patient woke up. Also, the patient reported dysesthesia when shaving. Clinical examination showed the patient’s inability to discriminate direction, pressure (Von Frey filaments) and sensibility (cold/hot) on the skin of the affected side.

**Figure 2:**
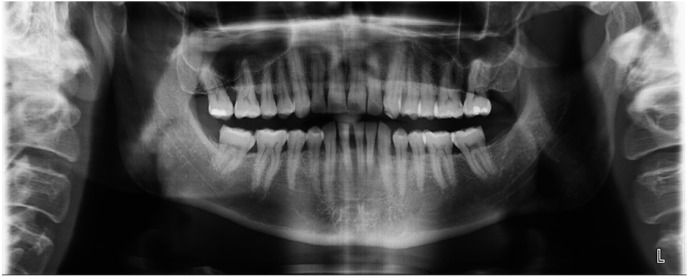
Orthopantomography made on the first visit. No abnormalities that may explain the neuropathic symptoms were observed.

It was diagnosed as idiopathic sensory neuropathy of the third branch of the fifth cranial nerve after an accurate clinical and radiographic evaluation (Fig. 2). Besides, local mialgia due to an eccentric bruxism was also diagnosed, which resulted in a widespread dental attrition. Physiotherapic treatment in combination with an oral splint (Michigan-type splint) were prescribed in order to reduce dental wear and trying to enhance muscle relaxation.

Patient reported a marked improvement in terms of the tingling sensation after three months of treatment, with a complete disappearance of the dysesthetic feeling that patient referred. Extraoral examination revealed pressure, direction and sensibility discrimination in the skin of the third quadrant. On the other hand, the left side of the tongue experienced a remarkable improvement as evidenced by pressure and direction testing, despite a slight residual tingling.

After 5 years of follow up, the tingling remained stable, being especially intense in the morning, without a complete remission of numbness. The clinical evolution confirms the diagnosis of buccal and lingual nerve neuropathy by muscle compression, which manifest itself as paresthesia in the territory innervated by these nerves.

## Discussion

Idiopathic sensory neuropathy of the trigeminal nerve is a benign disorder characterized clinically by facial numbness confined to the territory of one or more divisions of the trigeminal nerve, persisting from a few weeks to several years in which there is no identifiable underlying disease (6). Clinically it is classified into three groups: a primary acute form, chronic form associated with connective tissue diseases, and a chronic idiopathic form (6-8).

Several publications on this type of pathology are focused on clinical cases or small series of cases, making a diagnosis after excluding several diseases that can affect in a greater or lesser degree all three branches of the trigeminal nerve. It is mandatory to rule out tumor pathology, either local or metastatic (breast, prostate, lung, thyroid, liver and stomach), and collagen vascular or demyelinating disease (multiple sclerosis). ( Table 1) (6-9)

**Table 1:**
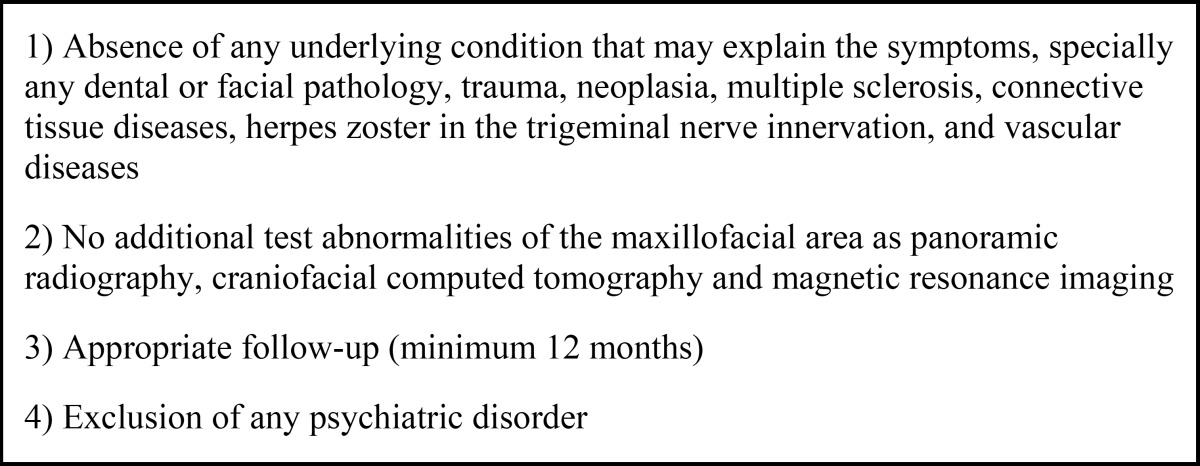
Exclusion criteria for the diagnosis of idiopathic sensory neuropathy of the trigeminal nerve.

Some authors state that this neuropathy is rarely accompanied by pain and has a rapid onset with a self-limiting course from weeks to years (7,8), associated in many cases to an autoimmune disorder (10) or viral infection (8). In our case the tingling sensation was accompanied initially by dysesthesia and all possible etiologic factors cited previously were ruled out

The way that follows the third branch of the trigeminal nerve is accurately described in the literatura. However, several studies performed in cadavers have detected anatomical abnormalities in their pathway that may be consider as etiological factor of certain neropathies as numbness or tingling, specially if they elapsed between muscle bundles or anatomical holes. (1-5,11). The buccal nerve, for example, passes through the two bundles of the pterygoid facing the risk of sensory alterations due to muscle contraction (11-14).

The territory innervated by the buccal nerve is controversial because of the difficulty of determining their boundaries. Tubbs *et al.* (12) performed the dissection of forty bodies setting the distance between the buccal nerve and the lip commissure as the only reference statistically significant for identifying the path. Buccal nerve injuries are associated mainly with the distal incision made during the surgical extraction of third molars, in orthognathic surgery and in facial trauma. However, perception of the disturbances of the buccal nerve is difficult to be accurately diagnosed because the areas innervated by the mental nerve or the infraorbital nerve overlap with the buccal nerve (14).

The buccal nerve is not the only nerve crossing the bundles of the masticatory muscles. Loughna *et al.* (3) in a study of 52 corpses found in three pieces of dissection that the posterior trunk of the mandibular nerve (lingual nerve, inferior alveolar and auriculotemporal) passed through the inferior fascicle of the lateral pterygoid muscle. Similarly, they observed that the mylohyoid nerve and anterior deep temporal nerve sometimes passed through the same muscle, concluding that this type of muscle entrapment in the infratemporal fossa allows anatomic and clinical relationships between the mandibular nerve and pterygoid muscle. These findings establish the hypothesis that a sustained spasm of this muscle can cause nerve compression that can manifest as tingling, pain or both in the areas of innervation.

Along the same line, Maeda *et al.* (13) established the diagnosis of paroxysmal type neuralgia of the bucal nerve in three patients. In all cases the pain radiated in the field of innervation of the ipsilateral buccal nerve, taking as a possible cause nerve compression by hyperactivity of the temporal muscle, due to its proximity to the buccal nerve at its insertion into the anterior and internal mandibular ramus. To support this assumption, 26 dissection of cadavers were carried out to study the anatomical relationship between these two structures, finding that in 6 (12%) of the 52 pieces, the buccal nerve crossed the temporal muscle in its lower insertion. These findings establish the hypothesis that the buccal nerve compression can cause a paroxysmal neuropathic unilateral pain in this orofacial region.

## Conclusions

Idiopathic sensory neuropathy of the trigeminal nerve is a benign disorder characterized clinically by facial numbness confined to the territory of one or more divisions of the trigeminal nerve. The diagnosis is made by exclusion after ruling out central and / or peripheral alterations and systemic disease. Cadaveric studies show the path that follow different branches of the trigeminal nerve through the masticatory muscles, being the lingual nerve and especially the buccal nerve those most affected. Hyperactivity of the fascicles of these muscles generates a compression that could explain certain orofacial neuropathies (numbness and / or pain) in which a clear etiologic factor can not be found.
